# Nanoparticle delivery of a pH-sensitive prodrug of doxorubicin and a mitochondrial targeting VES-H_8_R_8_ synergistically kill multi-drug resistant breast cancer cells

**DOI:** 10.1038/s41598-020-65450-x

**Published:** 2020-05-26

**Authors:** Petro Czupiel, Vianney Delplace, Molly Shoichet

**Affiliations:** 1 Department of Chemical Engineering and Applied Chemistry, University of Toronto, 200 College Street, Toronto, ON M5S 3E5 Canada; 2 Institute of Biomaterials and Biomedical Engineering, University of Toronto, 164 College Street, Toronto, ON M5S 3G9 Canada; 3 Donnelly Centre, University of Toronto, 160 College Street, Toronto, ON M5S 3E1 Canada

**Keywords:** Breast cancer, Drug delivery

## Abstract

Multi-drug resistance (MDR) remains a major obstacle in cancer treatment while being heavily dependent on mitochondrial activity and drug efflux. We previously demonstrated that cationic lipids, such as the vitamin E succinate modified octahistidine-octaarginine (VES-H_8_R_8_) conjugate, target mitochondria, resulting in depolarized mitochondria and inhibited drug efflux in MDR breast cancer cells. We hypothesized that the effective cell uptake, efflux inhibition, and mitochondrial depolarization properties of VES-H_8_R_8_ would synergistically enhance the toxicity of a pH-sensitive prodrug of doxorubicin (pDox) when co-encapsulated in nanoparticles (NPs). pDox was successfully synthesized and validated for pH-sensitive release from NPs under lysosome-mimicking, acidic conditions. The synergistic effect of VES-H_8_R_8_ and pDox was confirmed against MDR breast cancer cells *in vitro*. Importantly, synergism was only observed when VES-H_8_R_8_ and pDox were co-encapsulated in a single nanoparticulate system. The synergistic mechanism was investigated, confirming superior pDox uptake and retention, Pgp efflux inhibition, mitochondrial depolarization, and enhanced induction of ROS, and apoptosis. This work demonstrates the translational potential of doubly-loaded NPs co-encapsulating pDox with VES-H_8_R_8_ to synergistically kill MDR breast cancer cells.

## Introduction

Multi-drug resistance (MDR) remains a major obstacle in cancer treatment whereby MDR cancer cells are able to survive and proliferate under clinical doses of chemotherapeutic agents^1^. MDR can be intrinsic or acquired, and results in cross-resistance to multiple chemotherapeutics. Intrinsic MDR arises from hyperactive detoxification processes that counteract the mechanisms of action of chemotherapies such as oxidant scavenging or regulation of pro-apoptotic proteins^2,3^. Nonspecific acquired resistance arises when cancer cells overexpress drug efflux pumps, such as breast cancer resistance protein, multidrug resistance-associated protein, and permeation glycoprotein (Pgp)^4–6^. For example, Pgp expression in cancer tissue is increased from 11% in untreated patients to 30% in patients who underwent chemotherapy, resulting in an increased prevalence of MDR tumors^7^. Drug efflux is an active process, requiring adenosine triphosphate (ATP) for pumping various chemotherapeutics out of the cell, lowering the effective intracellular drug concentration^8^. Both intrinsic and acquired MDR are heavily dependent on the mitochondria, making the mitochondria of MDR cancer cells an attractive intracellular target.

Interestingly, the mitochondria of cancer cells are hyperpolarized, opening new opportunities for tumor targeting^2^. For example, Neu4145 cancer cells exhibit a mitochondria membrane potential of −210 mV, whereas the mitochondrial membrane potential of non-cancer cells ranges around −138 mV^9–11^. Furthermore, MDR cancer cells are able to retain active and polarized mitochondria following chemotherapy treatment, allowing electrophoretic targeting of MDR cancer cells^12^. Traditionally, delocalized lipophilic cations (DLC), such as triphenylphosphine- and guanidine-containing compounds (e.g., arginine), are used to electrophoretically target mitochondria. Mitochondrial-penetrating peptides, composed of alternated hydrophobic and cationic amino acids, have also been designed to deliver chemotherapeutics specifically to mitochondria, showing enhanced chemotherapeutic activity^13^. Most recently, we developed a vitamin E succinate modified octahisitidine-octaarginine (VES-H_8_R_8_) conjugate with similar structural features that targets cancer mitochondria and exhibits selective toxicity in MDR cancer cells^14^. VES-H_8_R_8_ was demonstrated to kill MDR cancer cells via mitochondria depolarization, induction of reactive oxygen species (ROS), apoptosis, and G1 cell cycle arrest. Importantly, VES-H_8_R_8_ demonstrated good cell uptake, retention and Pgp efflux inhibition in MDR cancer cells, which makes VES-H_8_R_8_ an ideal candidate for co-delivery with other chemotherapeutics, such as doxorubicin, for potential synergism.

Nanoparticles (NPs) carry many advantages, such as solubilization of hydrophobic therapeutics, passive tumor targeting via the enhanced permeability and retention effect, and, importantly, encapsulation of multiple therapeutics^15–17^. Moreover, relative to free drug combinations, drug co-encapsulation strategies offer novel and enhanced therapeutic advantages in the clinical setting^18^. Free drug combinations do not guarantee optimal drug ratios in diseased tissues as drugs typically have differing metabolism, pharmacokinetics, and tumor uptake. On the contrary, co-encapsulating drugs in NPs allows spatiotemporal control *in vivo*, wherein the initial drug ratio is maintained from the injection site to the tumor. For example, the co-encapsulation of doxorubicin and mitomycin C in NPs was therapeutically superior to free drug combination *in vivo*, with prolonged systemic circulation, enhanced tumor accumulation, and maintenance of synergistic drug ratios over 24 h^19^. The synergistic co-encapsulation of cytarabine and daunorubicin also demonstrated clinical success against high-risk acute myeloid leukemia (AML), and is the first nanotherapy in 40 years to improve the survival of patients with AML^20,21^. Thus, NPs are suitable carriers for multiple drugs and support synergistic anti-cancer activity against tumors.

**Figure 1:**
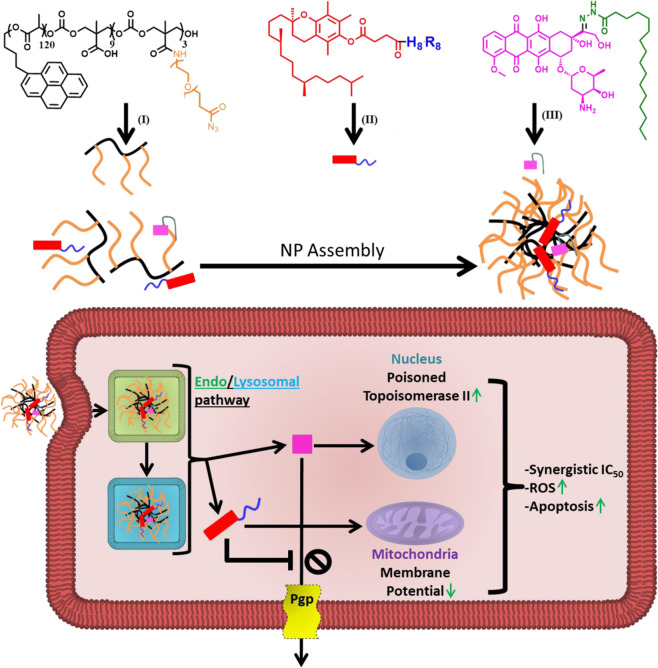
Schematic representation of the proposed mechanism of synergy of co-encapsulated vitamin E succinate modified octahistidine-octaarginine (VES-H_8_R_8_) and palmityl-doxorubicin (pDOX). (i) Structure of poly(D,L-lactide-co-2-methyl-2-carboxytrimethylenecarbonate)_12K_-g-poly(ethylene glycol)_10K_ (P(LA-*co*-TMCC)-*g*-PEG) used as amphiphilic polymer for NP synthesis. (ii) Structure of vitamin E succinate modified octahistidine-octaarginine (VES-H_8_R_8_), with vitamin E succinate in red and octahistidine-octaarginine in blue. (iii) Structure of the pH-responsive, palmityl-doxorubicin (pDOX), with doxorubicin in pink, palmityl moiety in green, and the pH-responsive hydrazone bond in black. VES-H_8_R_8_ and pDOX loaded NPs (VDNPs) enter cells through the endolysosomal pathway where VES-H_8_R_8_ and hydrolyzed pDOX escape into the cytosol. VES-H_8_R_8_ affects the mitochondria and decreases the mitochondrial membrane potential. VES-H_8_R_8_ inhibits the permeation glycoprotein (Pgp) efflux of Dox, allowing the cytosolic accumulation of Dox. Accumulated Dox poisons topoisomerase II and, together with VES-H_8_R_8_, synergistically kills multi-drug resistant breast cancer cells.

We explored, for the first time, the potential synergism of co-encapsulated VES-H_8_R_8_ and doxorubicin in an MDR breast cancer model. As doxorubicin hydrochloride is water soluble (>50 mg/mL) and rapidly leaks out of NPs, we envisioned to use a pH-sensitive, hydrophobic prodrug of doxorubicin that is suitable for encapsulation^22,23^. Using a hydrazone linkage, the palmityl-modified doxorubicin (pDox) is expected to be water-insoluble at neutral pH while releasing doxorubicin from NPs in the acidic endolysosomal environment upon cancer cell uptake^24,25^. We hypothesized that co-encapsulating VES-H_8_R_8_ with pDox in NPs would lead to synergism in MDR cancer cells (Fig. 1). To test this hypothesis, pDox was synthesized and tested for pH release in lysosome-mimicking, acidic medium when encapsulated in NPs. The synergistic co-encapsulation of VES-H_8_R_8_ and pDox was tested against both parental and MDR breast cancer cells^18^. The synergistic mechanism of VES-H_8_R_8_ and pDox was investigated through NP uptake and retention, Pgp efflux inhibition, mitochondria depolarization, induction of ROS, and cell death mechanism. This work demonstrates the synergistic mechanism of co-encapsulating a novel mitochondrial depolarizer and Pgp efflux inhibitor, VES-H_8_R_8_, with a pH-sensitive prodrug of doxorubicin in NPs against MDR cancer cells.

## Materials and Methods

All solvents and reagents were purchased from Sigma-Aldrich and used as received unless otherwise noted. Peptide synthesis reagents were purchased from AnaSpec (Fremont, CA).

### Vitamin E Succinate modified Octahistidine-Octaarginine (VES-H_8_R_8_) synthesis

VES-H_8_R_8_ was synthesized as previously reported^14^. Briefly, H_8_R_8_ was synthesized by conventional solid-phase microwave assisted peptide synthesis techniques (CEM Liberty 1) on a rink amide ProTide Resins were used (CEM Corp, NC, USA). The N-terminus of H_8_R_8_ was modified using 5.0 eq. of vitamin E succinate activated with 4.9 eq of 2-(6-Chloro-1-H-benzotriazole-1-yl)-1,1,3,3-tetramethylaminium hexafluorophosphate (HCTU) for 24 h. After washing the resin, the peptide was cleaved using TFA:H2O:TIS (95:2.5:2.5) for 5 hours and purified through a C18 reverse phase column (Silicycle, Quebec, Canada). Peptide mass was verified through Electrospray ionization or matrix assisted laser desorption ionization-time of flight mass spectrometry using the Agilent 6538 Q-TOF mass spectrometer.

### Palmityl-Doxorubicin synthesis

Palmitic acid hydrazide was reacted with the ketone of doxorubicin, to produce the ph-sensitive hydrazone bond containing palmityl-doxorubicin (pDox), as synthesized previously (Fig. S1A)^22,26^. Briefly, 150 mg of doxorubicin free base (MedKoo Biosciences, Inc, NC, USA) and 82 mg of Palmitic acid hydrazide (1.1 eq) were added to 300 mL of MeOH:DCM solution (1:1) containing 15 uL of trifluoroacetic acid. The reaction proceeded for 24 hours and reaction progress monitored by thin layer chromatography (TLC) using MeOH:DCM (1:3). After the reaction was complete, the solvent was removed by rotary evaporation, and the crude was dissolved in a small volume of MeOH:DCM (1:20). The dissolved crude was purified by silica column chromatography using DCM, and eluting with increasing concentrations of MeOH until the product, pDox, was eluted. Fractions containing pDox were collected, solvent removed by rotary evaporation, and placed in a vacuum dry oven to yield a red oil (yield = 65%). The ^1^H NMR spectrum of pDox exhibited chemical shifts of δ 10.01 (1H, s), 8.03 (1H, s), 7.78 (1H, s), 5.53–5.52(2H, d), 4.68 (2H, d), 3.91–4.09 (3H, m), 3.08–3.46 (6H, m), 2.03–2.60 (7H, m), 1.67–1.98 (8H, m), 1.02–1.42 (29H, m),0.85–0.88 (6H, m) ppm. (Fig. S1B) The molecular ion peak ([M + H]+) of pDox was 796.63. (Fig. S1C)

### Polymer synthesis

Poly(D,L-lactide-co-2-methyl-2-carboxytrimethylene carbonate)_12K_-graft poly(ethylene glycol)_10K_-azide (P(LA-*co*-TMCC)-*g*-PEG-N_3_) was synthesized following previously established protocols^1,2^. Briefly, a ring opening polymerization of D,L-lactide and 2-methyl-2-carboxy-trimethylenecarbonate catalyzed by a thiourea and initiated by a pyrenebutanol affords P(LA-*co*-TMCC) with a molecular weight of ~12,000 g/mol. 10,000 g/mol PEG chains are grafted onto the backbone by carbodiimide chemistry. There were ~2 PEGs/backbone as determined by H^1^ NMR.

### Nanoparticle preparation

20 wt% of VES-H_8_R_8_ and 25 wt% of pDox were added to 6 mg of P(LA-*co*-TMCC)-*g*-PEG-N_3_ in 100 uL of methanol:acetonitrile solution (1:1), and then diluted with 2 mL of dH_2_O. Singly loaded NPs were prepared using an identical NP protocol as before, except only one drug was added at the same wt%. Prepared NPs were filtered through a 0.45 µm um PES filter, and aliquots were frozen until required. The diameters and zeta potential of NPs were measured using a Malvern Zetasizer Nano ZS (4 mW, 633 nm laser) at a concentration of 1 mg/mL

### Drug loading characterization

NP formulations containing pDox were quantified by fluorescence using the Tecan Infinite M200 Pro fluorescent plate-reader. NP formulations were diluted in DMSO:PBS (200:25), and pDox encapsulation was quantified by fluorescence measurement (ex/em 480/580 nm) against a standard curve. VES-H_8_R_8_ encapsulation was quantified using amino acid analysis. Average drug loading was obtained from three separate NP batches, calculated as follows:

$$Drug\,Loading( \% )=\frac{Mas{s}_{pDox}+\,Mas{s}_{VHR}}{Mas{s}_{pDox}+\,Mas{s}_{VHR}+\,Mas{s}_{Polymer}\,}\times 100$$

### pDox release study

Doxorubicin control, singly loaded NPs, DNPs, or doubly loaded NPs, VDNPs, were diluted in either PBS (pH = 7.4) or PBS/citric acid (pH = 5.0) at a polymer concentration of 100 µ/mL and sealed in dialysis tubing (Spectra/Por 6, 3.5 kDa, Spectrum Labs, CA, USA). The formulations were then allowed to dialyze against 40 mL of either PBS (ph = 7.4) or PBS/citric acid (pH=5.0), under shaking at 37 °C. At indicated times, 25 uL of the dialysate was taken, diluted with 200 uL of DMSO before fluorescence measurement (ex/em: 480/580 nm) as a proxy for Dox release. After 48 h, the remaining encapsulated pDox was quantified as above. Three batches of each formulation were tested. Released Dox was calculated as follows:

$$Released\,Dox( \% )=\frac{Fluorescence\,Intensity\,of\,Do{x}_{DiasylateatTimeX}}{Fluorescence\,Intensity\,of\,pDo{x}_{Encapsulatedat48h}}\times 100$$

### Cell cultures

Both WT (EMT6/P) and doxorubicin resistant (EMT6/AR1) EMT6 cells were generously provided by Dr. X.Y. Wu at the University of Toronto, originally from Dr. Ian F. Tannock at the Ontario Cancer Institute, (Toronto, ON, Canada) and maintained in our laboratory. Non-cancerous fibroblast cells (NIH/3T3) were grown in DMEM supplemented with 10% fetal calf serum and 1% penicillin/streptomycin at 37 °C in a humidified incubator with 5% CO2 atmosphere. EMT6/P cells were grown in α-MEM supplemented with 10% fetal bovine serum and 1% penicillin/streptomycin at 37 °C in a humidified incubator with 5% CO2 atmosphere. EMT6/AR-1 cells were grown as EMT6/P, with the addition of doxorubicin at 1 ug/mL to maintain doxorubicin resistance and Pgp overexpression.

### NP potency assay

Cells were seeded into 96-well flat-bottomed tissue culture plates at a density of 3,000 cells per well, and allowed to adhere for 24 hours. Treatments were incubated with cells for 24 hours, then replenished with fresh media and incubated for an additional 48 hours. Singly-loaded NPs, DNPs and VNPs, were dose-matched to the respective drug concentration of doubly loaded NPs, VDNPs. Free doxorubicin was used as a control where doxorubicin hydrochloride was dissolved in dimethylsulfoxide (DMSO) at a concentration of 1 mg/mL. Free VES-H8R8 was also used as a control dissolved in DMSO at a concentration of 1 mg/mL. Free doxorubicin was used as a control where doxorubicin hydrochloride was dissolved in dimethylsulfoxide (DMSO) at a concentration of 1 mg/mL. Free VES-H_8_R_8_ was also used as a control dissolved in DMSO at a concentration of 1 mg/mL. After the addition of NP formulations, cells were allowed to grow for 24 h at 37 °C in a 5% CO_2_ and 95% air humidified incubator. The Presto Blue (Life Technology) assay was used as a proxy for cell relative viability, calculated as follows:

$${\rm{R}}{\rm{e}}{\rm{l}}{\rm{a}}{\rm{t}}{\rm{i}}{\rm{v}}{\rm{e}}\,{\rm{V}}{\rm{i}}{\rm{a}}{\rm{b}}{\rm{i}}{\rm{l}}{\rm{i}}{\rm{t}}{\rm{y}}({\rm{ \% }})=\frac{{\rm{F}}{\rm{l}}{\rm{u}}{\rm{o}}{\rm{r}}{\rm{e}}{\rm{s}}{\rm{c}}{\rm{e}}{\rm{n}}{\rm{c}}{\rm{e}}\,{{\rm{I}}{\rm{n}}{\rm{t}}{\rm{e}}{\rm{n}}{\rm{s}}{\rm{i}}{\rm{t}}{\rm{y}}}_{Treated}}{{\rm{F}}{\rm{l}}{\rm{u}}{\rm{o}}{\rm{r}}{\rm{e}}{\rm{s}}{\rm{c}}{\rm{e}}{\rm{n}}{\rm{c}}{\rm{e}}\,{{\rm{I}}{\rm{n}}{\rm{t}}{\rm{e}}{\rm{n}}{\rm{s}}{\rm{i}}{\rm{t}}{\rm{y}}}_{Untreated}}\times 100{\rm{ \% }}$$

### Synergism analysis

Combination indices (CI) were calculated using the median-effect analysis taking advantage of the Compusyn software (ComboSyn Inc. NY, USA)^27,28^. The median-effect analysis considers both the shape and potency of the dose response curves on viability. The dose response curves of individual and combination treatments were linearized into a median effect plot of log[(f_a_)^−1^ − 1]^−1^ vs log[D], where f_a_ is the fraction of dead cells, and D represents the drug concentration. CI values >1, =1, <1 are indicative of antagonism, additive effects, and synergism, respectively.

### Confocal microscopy

EMT6/AR-1 cells were seeded at a density of 20,000 cells per well in a 8-well Nunc Lab-Tek II chambered cover glass (Thermo Fisher Scientific, MA, USA) and allowed to adhere for 24 hrs at 37 °C in a 5% CO2 and 95% air humidified incubator. The concentration of doxorubicin or pDox was kept at 5 µM for doxorubicin control, DNPSs and VDNPSs. After incubation times of 2, 5 and 24 hours, cells were washed 3x with PBS, and Hoescht 33342 nuclei acid dye (Molecular Probes, Inc., Eugene, OR, USA) in PBS was added. Live cell imaging was performed using an Olympus FV1000 confocal microscope equipped with an oil immersion 60x lens. Excitation and emission wavelengths are as follows: for Hoescht 33342, excitation at 405 nm, and emission at 460 nm, and for doxorubicin, excitation at 488 nm, and emission at 550 nm.

### Doxorubicin and pDox uptake and retention studies

20,000 cells were seeded into 48-well plates and allowed to adhere for 24 h at 37 °C in a 5% CO2 and 95% air humidified incubator. The concentration of doxorubicin or pDox was kept at 5 µM for doxorubicin control, DNPs and VDNPs. DNPs were either incubated alone, or with VNPs dose-matched at the concentration of VES-H_8_R_8_ in VDNPs. To test Pgp inhibition, DNPs were also incubated with 20 µM of vitamin E succinate. Untreated cells were used as negative control to determine background. After 24 hours of treatment, cells were washed thrice with PBS, and harvested with trypsin. Cell fluorescence was analyzed using a BD Accuri C6 flow. Cell debris and doublets were gated out using FSH-A vs FSH-H, and at least 10,000 events were collected. The mean fluorescence intensity was measured on three biological repeats to evaluate the doxorubicin or pDox uptake and retention.

### Mitochondrial membrane polarization assay

Mitochondrial membrane potential was assayed using the JC-1 probe (5,5′,6,6′-tetrachloro-1,1′,3,3′-tetraethyllenzimi dazolycarbocyanine iodide) (Biotium Inc., CA, USA). Both DNP- and VDNP-treated cells exhibited fluorescence in the FL-2 channel due to the fluorescence of pDox. As such, VNPs were used to explore the effects of mitochondrial membrane polarization. VNPs was incubated at its IC_50_ (14 µM) as well as the IC_50_ of the concentration of VES-H_8_R_8_ in VDNPs (4 µM). 20,000 cells were seeded into 48-well plates and allowed to adhere for 24 h at 37 °C in a 5% CO2 and 95% air humidified incubator. The cells were treated the next day for 5 hours in full media. Carbonyl cyanide m-chlorophenyl hydrazone (CCCP) is a rapid mitochondrial depolarizer and was used as a positive control (50 µM). Following treatment, the cells were washed with PBS thrice, and then incubated at 37 °C with 10 µM of JC-1 in full media for 30 min. The cells were then washed thrice in PBS, harvested, and placed on ice before measuring fluorescence in a flow cytometer within an hour. Cell debris and doublets were gated out using FSH-A vs FSH-H, and at least 10,000 events were collected. A gate was set according to DMSO and CCCP treated cells in the FL-2 channel (585 nm / 40 nm) to measure the proportion of JC-1 aggregate fluorescence versus JC-1 monomer fluorescence in the FL-1 channel (533 nm / 30 mn). Averages were obtained from three biological repeats. Changes in the mitochondria membrane potential (ΔΨm) were expressed using the following equation:

$$Relative\,Mitochondrial\,Membrane\,Potential=\frac{\frac{JC-{1}_{aggregate}}{JC-{1}_{monomerTreatment}}}{\frac{JC-{1}_{aggregate}}{JC-{1}_{monomerControl}}}\times 100 \% $$

### ROS production assay

Reactive oxygen species (ROS) in cells was detected using the cellular reactive oxygen species detection assay kit (ROS Deep Red) (abcam, ON, Canada) as per manufacturers protocols. 20,000 cells were seeded into 48-well plates and allowed to adhere for 24 h at 37 °C in a 5% CO2 and 95% air humidified incubator. The cells were treated the next day for 2, 5, or 24 hours at the IC_50_ of VDNPs, where DNPs, VNPs, and DNPs+VNPSs were dose-matched to the respective concentration of pDox or VES-H_8_R_8_ in VDNPs. Untreated control cells were used to measure background fluorescence in the FL-4 channel. Hydrogen peroxide (H2O2) at 1 mM was used as a positive control. Following treatment, the cells were washed with PBS thrice, and then the cells were incubated at 37 C in full media containing ROS deep red for 30 min. The cells were washed 3x with PBS, harvested, and placed on ice before measuring fluorescence in a flow cytometer within an hour. Cell debris and doublets were gated out using FSH-A vs FSH-H, and 10,000 events were collected. The mean fluorescence intensity was collected in the FL-4 channel (575 nm/25 nm) and averaged from three biological repeats. The results were expressed as fold increase in mean fluorescence intensity of treated cells relative to the untreated control group.

### Apoptosis and necrosis assay

Apoptosis was measured using Annexin V-Cy5 (Biovision Inc, CA, USA) and Live or Dead Fixable Dead Cell Staining Kit [Deep Red Fluorescence] (Catalog # 22604, AAT Bioquest, CA USA). Apoptotic cells have exposed phosphatidylserine on the external cell membrane that binds to Annexin V-Cy5, while necrotic cells exhibit membrane damage that allows Live or Dead Fixable Dead Cell Staining dye to bind to DNA. Briefly, 20,000 EMT6/AR-1 cells were seeded into 48-well plates and allowed to adhere for 24 h at 37 °C in a 5% CO2 and 95% air humidified incubator. To analyze apoptosis, the EMT6/AR-1 cells were treated the next day for 2, 5, or 24 hours at the IC_50_ of VDNPs, where DNPs, VNPs, and DNPs+VNPs were dose-matched to the respective concentration of pDox or VES-H_8_R_8_ in VDNPs. Untreated control cells were used to measure background fluorescence in the FL-4 channel. Identical setup for the apoptosis assay was used to analyze necrotic cells, but only for a 24 hour incubation. Following treatment, floating cells were collected, adhered cells were harvested, washed with PBS, and then incubated with Annexin V-Cy5 (1 uL/1 mL) or with Live or Dead Fixable Dead Cell Staining Kit for 20 minutes at 25 °C as per manufacturer’s protocol. Cells were then placed on ice before measuring fluorescence in a flow cytometer within an hour. Cell debris and doublets were gated out using FSH-A vs FSH-H, and at least 10,000 events were collected. Gates were set in the FL-4 channel (ex = 640 nm, em = 675 nm / 25 nm) for Annexin-V-Cy5 or Live or Dead Fixable Dead Cell Staining relative to unstained controls. Proportion of apoptotic or necrotic cells were averaged from 3 biological repeats.

### Statistical analysis

All statistical analyses were performed using Graph Pad Prism version 6.00 for Windows (Graph Pad Software, San Diego, California, www.graphpad.com). Differences among groups were assessed by one-way ANOVA with Bonferroni post hoc correction. Graphs are annotated where p-values are represented as *p ≤ 0.05, **p ≤ 0.01, or ***p ≤ 0.001. All data are presented as mean ± standard deviation.

## Results and Discussion

### NP characterization and pDox release

Blank NPs, singly loaded NPs, VES-H_8_R_8_-NPs (VNPs) and pDox-NPs (DNPs), and doubly loaded NPs, VES-H_8_R_8_-pDox-NPs (VDNPs) were prepared by nanoprecipitation in water. The average hydrodynamic sizes of the NPs were ~60 nm, indicating that the size of the NPs is independent of drug encapsulation. (Table 1) Most of the NPs also exhibited polydispersity indices below 0.20, indicative of narrow size distribution^29^. The zeta potential of blank NPs was −12.9 ± 1.6 mV, while the zeta potential of singly loaded NPs were −4.6 ± 0.7 mV and 11.8 ± 1.8 mV for DNPs and VNPs, respectively. VDNPs, exhibited a zeta potential of 13.6 ± 1.7 mV. While all the values are in the range of neutral zeta potential (−20 to +20 mV), the trends from negative to positive charge is indicative of successful encapsulation of cationic VES-H_8_R_8_ and pDox^30,31^. The more neutral zeta potential of DNPs is attributed to the protonated amine of pDox neutralizing the free acids of P(LA-*co*-TMCC)-*g*-PEG, while the more positive zeta potential of VDNPs is attributed to positive charges found within both pDox and VES-H_8_R_8_. The critical micelle concentration (CMC) of blank NPs and VNPs were 0.66 ± 0.12 µM and 1.25 ± 0.35 µM, respectively, in agreement with the CMC previously reported^29,32^. DNPs achieved a pDox loading of 13.4 ± 1.6%, while VNPs achieved a VES-H_8_R_8_ loading of 15.1 ± 0.9%. Interestingly, while pDox alone precipitates, encapsulated pDox is soluble (Fig. S2A), indicating successful drug encapsulation. VDNPs achieved similar drugs loadings to each singly-loaded NPs, with pDox loading of 10.9 ± 1.3% and VES-H_8_R_8_ loading of 11.7 ± 0.7%, while the total drug loading amounts to 22.6%. The concentration of each drug in the encapsulated NPs is shown in Fig. S2B.

**Table 1:** Physicochemical characteristics of blank NPs, and singly and doubly loaded NPs.

Name	Size [d.nm]^a^	PDI^b^	Zeta Potential (mV)^c^	Critical Micelle Concentration (µM)^d^	pDox Loading (%)^e^	VES-H_8_R_8_ Loading (%)^f^
NP	64 ± 9	0.19 ± 0.04	−12.9 ± 1.6	0.66 ± 0.12		
DNPs	59 ± 3	0.20 ± 0.02	−4.6 ± 0.7		13.4 ± 1.6	
VNPs	57 ± 3	0.21 ± 0.02	11.8 ± 1.8	1.25 ± 0.35		15.1 ± 0.9
VDNPs	68 ± 6	0.19 ± 0.01	13.6 ± 1.7		10.9 ± 1.3	11.7 ± 0.7

^a^Dynamic Light Scattering (DLS).

^b^Polydispersity index (PDI) determined by DLS.

^c^Zeta potential measurement calculated with NPs in water at 1 mg/mL.

^d^Pyrene Method.

^e^Doxorubicin Fluorescence in 10:1 DMSO:PBS against a standard curve, ^f^Amino Acid Analysis.

**Figure 2:**
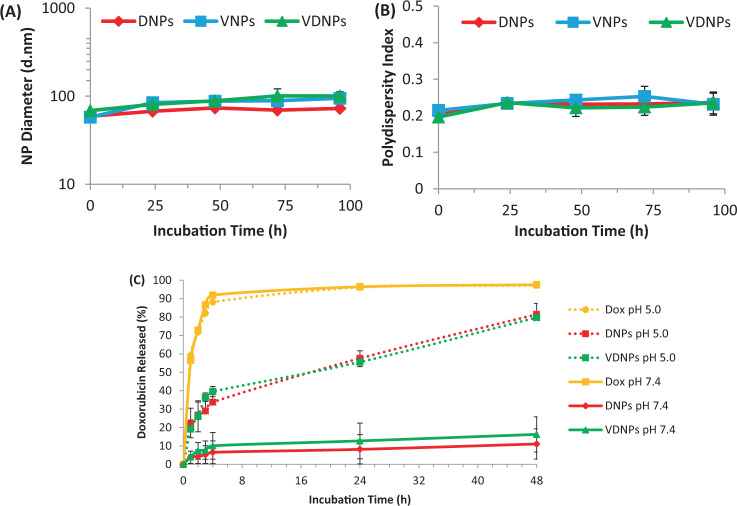
Nanoparticle (NP) stability studies and pH-responsive release of palmityl-doxorubicin (pDox). Singly loaded NPs, pDox-NPs (DNPs) and vitamin E succinate modified octahistidine-octaarginine-NPs (VNPs), and doubly loaded NPs, vitamin E succinate modified octahistidine-octaarginine -pDox-NPS (VDNPs), were incubated in PBS at 37 °C and NP size (**A**) and polydispersity index (**B**) were measured every 24 hours. (**C**) pH-responsive release of pDOX loaded in singly loaded NPs, DNPs, or doubly loaded NPS, VDNPs, at pH 7.4 vs 5.0. Free doxorubicin (Dox) was used as a dialysis control. Performed through a dialysis test at 37 °C for the indicate times. Unmodified doxorubicin was used as control.

The NP stability was monitored by hydrodynamic size and polydispersity indices (PDI) measurements over time (Fig. 2A,B). Interestingly, while NPs with high drug loadings typically exhibit limited stability under physiological conditions, all formulations maintained size and PDI over at least 96 h at 37 °C^33^. These results show that these P(LA-co-TMCC)-g-PEG NPs are stable *in vitro* and should be efficacious *in vivo* as they have been previously shown to be stable in serum, with a half-life of 71 ± 12 h^29^, and to enhance tumour accumulation of chemotherapeutics vs. that with conventional excipient delivery^34^. The pH-dependent release of doxorubicin (Dox) from the pDox prodrug was validated by mass spectrometry, where all of the pDox was hydrolyzed within 24 h at pH 5.0 (Fig. S3). Dox release was studied at both physiological and acidic pH, using a dialysis test as previously reported^35,36^. Free Dox control showed the expected rapid release, with >85% Dox released within 4 h at both pH 5.0 and 7.4 (Fig. 2C). Both DNPs and VDNPs showed limited Dox release (<20%) over 48 h at pH 7.4. However, the same formulations released up to 81% of Dox under acidic conditions (pH 5.0). Together, these results confirm the pH-sensitive release of pDox from the NPs. The similar results obtained for DNPs and VDNPs also indicate that VES-H_8_R_8_ co-encapsulation do not influence pDox hydrolysis and subsequent Dox release. More importantly, the release study at pH 5.0 is representative of late lysosomal acidic conditions, suggesting suitable endolysosomal escape of Dox following cell uptake^36^.

### VDNPs synergistically increase anti-cancer activity in a mdr breast cancer model

The anti-cancer activity of pDox and VES-H_8_R_8_ were assessed in the parental breast cancer cell line, EMT6/P, and the MDR breast cancer cell line, EMT6/AR-1, using the Presto Blue metabolic assay. The ability of treated cancer cells to metabolize Presto Blue was a proxy for anti-cancer activity (Tables 2, S1). pDox alone could not be tested at concentrations greater than 20 µM because of limited solubility. Importantly, the pH-responsive release of doxorubicin from pDox was demonstrated at pH 5.0 (Fig. S3). This supports the pH-responsive release of doxorubicin when pDox encapsulated NPs get trafficked through the endo-lysosomal pathway. While the half maximal inhibitory concentration (IC_50_) of Dox in EMT6/P is 0.16 ± 0.02 µM, it increases up to 82.19 ± 7.36 µM in EMT6/AR-1, indicative of Dox efflux and MDR status in agreement with the literature^37,38^. The IC_50_ of VES-H_8_R_8_ in EMT6/AR-1 and EMT6/P was 12.02 ± 1.94 µM and 11.12 ± 2.56 µM, respectively. The IC_50_ of VES-H_8_R_8_ on the parental and that on MDR breast cancer cell lines were not significantly different, suggesting that the cell uptake and the mechanism of cancer cell death of VES-H_8_R_8_ are independent of the Pgp efflux. We also investigated the anti-cancer activity of the combination of free Dox and VES-H_8_R_8_ in both breast cancer cell lines. The molar ratio of Dox to VES-H_8_R_8_ was based on the drug loading of pDox and VES-H_8_R_8_ in NPs (Table 1). The combination of free Dox and VES-H_8_R_8_ in EMT6/P resulted in an IC_50_ with a total drug concentration of 0.30 ± 0.03 µM, similar to that of Dox alone (0.16 ± 0.02 µM), indicating that VES-H_8_R_8_ did not increase the anti-cancer activity of Dox in the drug-sensitive cancer cell line. On the contrary, the combination of free Dox and VES-H_8_R_8_ in the MDR EMT6/AR-1 cells resulted in an IC_50_ of 32.64 ± 1.70 µM requiring concentrations of 3.9-fold less Dox than Dox alone and 1.54-fold less VES-H_8_R_8_ than VES-H_8_R_8_ alone.

**Table 2:** IC_50_ and combination indices of free drugs and drug-loaded NPs on the multi-drug resistant breast cancer cell line, EMT6/AR-1.

Name	Dox: VES-H_8_R_8_ Molar Ratio^a^	IC_50_ (µM, Total drug)^b^	Combination Index^c^
Dox	0: 1	82.19 ± 7.36	
VES-H_8_R_8_	1: 0	12.02 ± 1.94	
Dox+ VES-H_8_R_8_	1: 0.37	28.81 ± 2.87	0.60 ± 0.17
DNPs	1: 0	39.40 ± 1.04	
VNPs	0: 1	14.00 ± 0.75	
DNPs + VNPs	1: 0.37	26.30 ± 2.30	0.99 ± 0.07
VDNPs	1: 0.37	16.09 ± 0.71	0.61 ± 0.04

^a^[Dox] and [VES-H_8_R_8_] determined by fluorescence measurements and amino acid analysis, respectively.

^b^Obtained from Presto Blue analysis of drug treated cells.

^c^Calculated using CompuSyn.

Next, we investigated the benefits of co-encapsulating VES-H_8_R_8_ and pDox in NPs. VNPs exhibited an IC_50_ in the low µM range in both EMT6/AR-1 and EMT6/P cells, similar to the anti-cancer activity of free VES-H_8_R_8_ (Table 2 and Fig. S1). Here, the total drug concentration is the sum of the concentrations of pDox and Ves-H_8_R_8_, as the polymers used in the NPs were previously shown to be non-toxic to cancer cells^39^. On the Dox-sensitive EMT6/P cells, DNPs, the mixture of DNPs and VNPs, and VDNPs exhibited IC_50_ in the range of 0.7–1.0 µM, attributed primarily to the anti-cancer activity of Dox. Here, co-encapsulating VES-H_8_R_8_ and pDox had no significant benefit against the parental cell line as the IC_50_ of VDNPs was similar to DNPs and the mixture of DNPs and VNPs. On the contrary, co-encapsulating VES-H_8_R_8_ and pDox improved treatment against the EMT6/AR-1 cells. The IC_50_ of DNPs and VNPs in EMT6/AR-1 was 39.4 ± 1.04 µM and 14.00 ± 0.75 µM, respectively. The mixing of DNPs and VNPs resulted in an IC_50_ with a total drug concentration of 26.30 ± 2.30 µM ([pDox] = 19.2 µM, [Ves-H_8_R_8_] = 7.1 µM), while VDNPs exhibited an IC_50_ with a total drug concentration of 16.09 ± 0.71 µM ([pDox] = 11.7 µM, [Ves-H_8_R_8_] = 4.3 µM), indicating that co-encapsulation is required for more potent anti-cancer activity. The anti-cancer activity of Dox was enhanced when encapsulated in NPs, with an IC_50_ 2.1-fold lower than free Dox alone. This observation is in line with the literature where drug-loaded NPs facilitate a higher intracellular concentration of the drug in MDR cancer cells by bypassing Pgp efflux, thereby exhibiting a lower IC_50_^38,40,41^. The requirement of co-encapsulation is fundamental for *in vivo* translation as co-encapsulated drug nanoformulations administered *in vivo* can lead to superior tumor reduction and maintenance of drug ratios at the tumor site relative to the co-administered free drugs^19^.

To assess the synergistic advantage of co-encapsulating VES-H_8_R_8_ and pDox in NPs, a combination index (CI) was calculated based on the median effect analysis^27^. A median effect plot was produced by converting the dose response curves in Fig. S2C,D into log [(fa)^−1^ − 1]^−1^ vs log (D), where fa is the fraction of non-viable cells and D is the drug concentration (Fig. S3). R^2^ > 0.90 indicates statistical validity of the analysis and conforms to mass action law^28^. Furthermore, nonparallel lines in the median effect plots infers non-exclusivity in the mechanism of action of VES-H_8_R_8_ and pDox^28^. The median effect plot was further analyzed to obtain a CI. CI > 1 reflects drugs antagonism, CI = 1 reflects drug additive effect, and CI < 1 shows drug synergism^27^. The CI of the mixture of DNPs and VNPs was 0.99 ± 0.07, indicating that the mixture of DNPs and VNPs was additive in anti-cancer activity. Importantly, the CI of VDNPs was 0.61 ± 0.04, suggesting synergism of the co-encapsulated drugs against the MDR EMT6/AR-1 cells. In previous studies, a similar CI was achieved with NP formulations combining cytarabine and daunorubicin at a molar ratio of 5:1, which demonstrated improved survival of patients^20,42^. The synergism of VDNPs may be attributed to the pH-dependent release of Dox from NPs, which would poison the topoisomerase II enzyme, coupled with the toxicity of VES-H_8_R_8_ by targeting of the mitochondria with subsequent depolarization^14,43,44^. Furthermore, the dose reduction index indicates the fold decrease of a drug required to achieve the IC_50_ in the combined treatment. The dose reduction index of pDox in VDNPs was 3.35 ± 0.23, indicating that a ~3.4 fold lower dose of pDox relative to DNPs was required to achieve the IC_50_. Similarly, the dose reduction index of VES-H_8_R_8_ indicates that a ~3.3 fold lower dose of VES-H_8_R_8_ relative to VNPs is required to achieve the IC_50_ of VDNPs. The reduced doses of both drugs would be beneficial *in vivo* where cardiotoxicity limits the maximum tolerated dose of single agent regimes^43^.

### VDNPs enhances uptake, and retention of pDox

**Figure 3:**
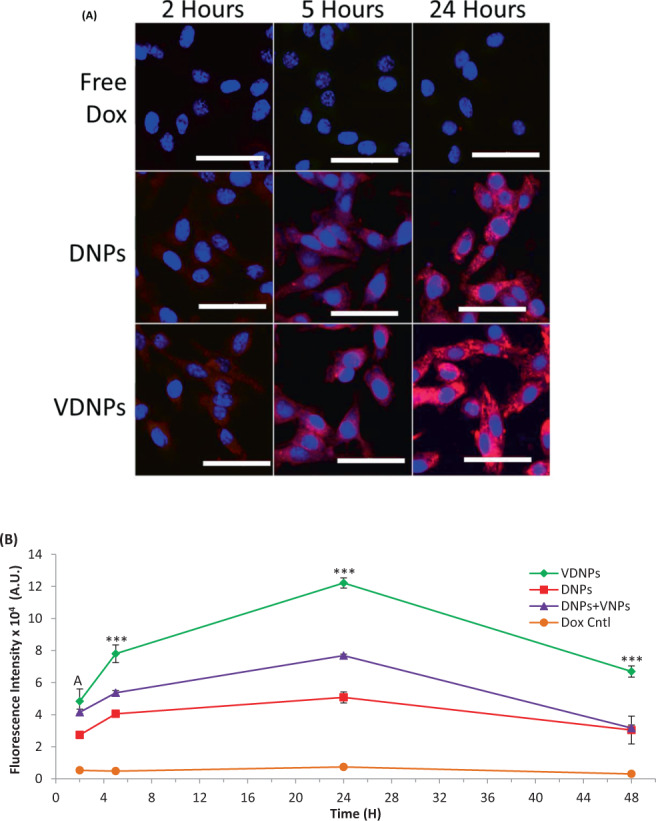
Multi-drug resistant (MDR) breast cancer cells treated with doubly loaded nanoparticles (NPs) show a significant increase in doxorubicin (Dox) accumulation and retention compared to controls. (**A**) Free Dox, singly loaded NPs, palmityl-doxorubicin-NPs (DNPs) and doubly loaded NPs, vitamin E succinate modified octahistidine-octaarginine-palmityl-doxorubicin-NPs (VDNPs), were incubated with MDR breast cancer cells for 2, 5, 24 h. Images were obtained by confocal microscopy and nuclei were stained with Hoechst 3342 and are shown in blue, and pDox or Dox is shown in red. (**B**) Time-dependent quantification of the uptake of Dox in MDR breast cancer cells with either VDNPs, DNPs alone, DNPs mixed with vitamin E succinate modified octahistidine-octaarginine-NPs (VNPs), and doxorubicin (Dox) control. Treatments were incubated for up to 24 h, followed by 24 h in fresh medium, prior to pDox/Dox fluorescence quantification. Data are presented as a mean (n=3) ± SD and statistical analysis was performed using one-way ANOVA and Tukey’s multiple comparison test (*** <0.001). A indicates significance of VDNPs and DNPs+VNPs against DNPs and Dox Control.

To elucidate the synergistic mechanism of VDNPs, we investigated the time-dependent uptake of free Dox, DNPs and VDNPs in MDR EMT6/AR-1 cells over 2, 5, and 24 h, using confocal microscopy (Fig. 3A). In EMT6/AR-1 cells, the free Dox control showed minimal Dox fluorescence at all time points, confirming active drug efflux in MDR cancer cells. Cells treated with DNP and VDNP exhibited both punctate organelles and fluorescence in the cytosol and nucleus at 24 h after treatment, indicating that the NPs were in the endo-lysosomal compartments and the doxorubicin was in the cytosol and the nucleus, which is consistent with previous reports^45^. Both DNPs and VDNPs showed time-dependent Dox accumulation up to 24 h post treatment. More importantly, VDNPs qualitatively exhibited enhanced uptake relative to DNPs and free Dox at all time points. DNP- and VDNP-treated cells exhibit punctate organelles, indicative of the endo/lysosomal entrapment, as well as diffuse cytoplasmic fluorescence, indicative of endosomal escape^46^. For both DNPs and VDNPs, there were indications of Dox release from the NPs and colocalization with cell nuclei, the intracellular target of Dox^44^.

We further quantified the difference in Dox fluorescence between free Dox, DNPs and VDNPs using flow cytometry (Fig. 3B). Here, we incubated treatment groups for up to 24 h to measure time-dependent uptake. To measure retention, we incubated treatment groups for 24 h, then replenished with fresh media and incubated the cells for an additional 24 h to measure the amount of Dox retained after 48 h. At all time points, VDNPs delivered significantly more Dox in the MDR EMT6/AR-1 cells relative to DNPs and free Dox controls (*** <0.001). Specifically, after a 24 h incubation, DNPs treated cells exhibited a mean fluorescence intensity (M.F.I.) of 50,823 ± 3,043, whereas VDNP-treated cells exhibited an M.F.I. of 122,192 ± 3,219, corresponding to a 2.4-fold increase in M.F.I when co-encapsulated. As expected, free Dox treated cells exhibited minimal fluorescence, indicative of active Dox efflux. We further validated the significance of co-encapsulating VES-H_8_R_8_ and pDox in NPs by comparing the uptake and retention of VDNPs to those of the mixture of DNPs and VNPs. DNPs were mixed with VNPS at identical molar ratio of pDox and VES-H_8_R_8_ to that of VDNPs. Co-incubation of DNPs and VNPs led to significantly more Dox retained in the MDR EMT6/AR-1 cells relative to DNPs at all time points (*** <0.001). Specifically, the M.F.I. of the mixture of DNP- and VNP-treated cells at 24 h was 76,872 ± 950, equivalent to 1.5 fold greater Dox retention in cells compared to DNPs alone, indicating that VNPs significantly inhibits Dox efflux and could be used as a MDR sensitizer. However, VDNP-treated cells at 24 h exhibited 1.6-fold greater Dox retention relative to the mixture of DNPs and VNPs. The slightly more positive surface charge of VDNPs enhances cell uptake and more Dox is delivered, as positively charged NPs exhibit increased cell penetration^47^. Importantly, the amount of Dox retained in VDNP-treated cells after 48 h is significantly increased compared to free Dox, DNPs, and the mixture of DNPs and VNPs. (*** <0.001) Here, VDNP-treated cells retained 2.1 fold more Dox than DNPs and the mixture of DNPs and VNPs. The increased M.F.I. observed with VDNPs may be attributed to the increased cell penetration in the presence of VES-H_8_R_8_. However, VES-H_8_R_8_ comprises vitamin E succinate that is an established Pgp efflux inhibitor, and VES-H_8_R_8_ was previously demonstrated to inhibit Pgp efflux in the MDR EMT6/AR-1 cells^14,48^. Therefore, we hypothesized that encapsulated VES-H_8_R_8_ inhibits the Pgp efflux of Dox, leading to increased Dox accumulation in the cytosol. To test our hypothesis, we incubated DNPs with free vitamin E succinate and evaluated Dox retention. After 24 h of incubation, followed by 24 h in fresh medium, the treated EMT6/AR-1 cells exhibited a 2.0 fold increase in Dox retention relative to DNPs alone at 48 h (Data not shown). The enhanced uptake and retention of VDNPs clearly demonstrate the benefits of co-encapsulating pDox and VES-H_8_R_8_. Improved uptake and retention along with Pgp efflux inhibition help to explain the synergism observed in the anti-cancer activity of VDNPs.

### Doubly loaded NPs induce mitochondrial depolarization and ROS

**Figure 4:**
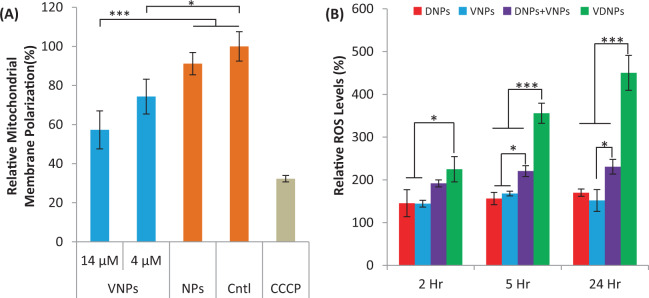
NPs containing vitamin E succinate modified octahistidine-octaarginine (VNPs) depolarizes the mitochondrial membrane, and vitamin E succinate modified octahistidine-octaarginine-palmityl-doxorubicin-NPs (VDNPs)induce ROS in a time-dependent manner in multi-drug resistant (MDR) breast cancer. (**A**) Relative mitochondria polarization of EMT6/AR-1 cancer cells treated with VNPs at the IC_50_ of VNPs (14 µM) or treated at the IC_50_ of VES-H_8_R_8_ in VDNPs(4 µM). Negative controls include no treatment (Cntl) and blank nanoparticles (NPs) (orange bars). The positive control, Carbonyl cyanide m-chlorophenyl hydrazone (CCCP), decreased the mitochondria polarization. (**B**) VDNPs treatment induces more ROS relative to singly-loaded NPs, palmityldoxorubicin-NPs (DNPs) and VNPs, or a mixture of singly loaded NPs, VNPs+DNPs, in a time-dependent manner. VDNPs was incubated at its IC_50_, where the other NP formulations were dose-matched to the respective drug concentration for either palmityl-Doxorubicin (pDox) or Vitamin E succinate modified octahistidine-octaarginine (VES-H_8_R_8_). Representative flow cytometry images can be found in Fig. S6. Data are presented as a mean (n = 3) ± SD and statistical analysis was performed using one-way ANOVA and Tukey’s multiple comparison test (* <0.05, *** <0.001).

To deepen the understanding of the synergistic mechanism of VDNPs, we confirmed the effect of VES-H_8_R_8_ on mitochondrial membrane potential when encapsulated in NPs. To monitor mitochondrial membrane potential, JC-1 was used as a mitochondria-specific polarization probe. To avoid Dox fluorescence interference with the JC-1 assay, we used VES-H_8_R_8_ loaded NPs. Mitochondrial membrane polarization data are shown normalized to no treatment group (Fig. 4A). As expected, blank NPs had no effect on mitochondrial membrane polarization, and the positive control group, carbonyl cyanide m-chlorophenyl hydrazone (CCCP), depolarized the mitochondrial membrane. At the IC_50_ concentration (14 µM), VNPs significantly depolarized the mitochondrial membrane relative to blank NPs and no treatment control, validating that VES-H_8_R_8_ is capable of mitochondrial depolarization when encapsulated in NPs (*** <0.001). More importantly, VNPs significantly depolarized the mitochondria at 4 µM, the equivalent dose of VES-H_8_R_8_ at the IC_50_ of VDNPs, relative to the no treatment group, indicating that depolarization is involved in the synergistic anti-cancer activity of VDNPs (* <0.05). These results are consistent with the mitochondrial depolarization induced by VES-H_8_R_8_^14^. Dox has also been demonstrated to indirectly depolarize cancer cell mitochondria through Dox-induced oxidative stress^49–51^. It is possible that the co-delivery of VES-H_8_R_8_ and pDox in VDNPs requires lower doses of each drug to affect mitochondria. VES-H_8_R_8_ may directly act upon the mitochondria while Dox induces intracellular events, such as reactive oxygen species (ROS) production, that indirectly affect mitochondria. The combined effects of encapsulated VES-H_8_R_8_ and pDox in VDNPs on mitochondrial depolarization further aid in understanding the synergistic cytotoxicity observed.

As both Dox and VES-H_8_R_8_ have been demonstrated to induce ROS, we investigated the effect of VDNPs on ROS levels, at the IC_50_ of VDNPs. As controls, the singly-loaded NPs and the mixture of singly loaded NPs were dose-matched to the respective drug in VDNPs. VDNPs significantly induced ROS at all time points (Fig. 4B). Specifically, VDNPs increased ROS levels to 224.9 ± 29.6%, 356.0 ± 23.5%, and 450.4 ± 40.8% relative to the no treatment group, upon incubation at 2, 5, and 24 h, respectively. Both DNPs and VNPs controls did not significantly induce an increase in ROS levels compared to no treatment. VDNPs significantly induced ROS levels compared to the mixture of DNPs and VNPs at 6 and 24 h of incubation, further highlighting the requirement of co-encapsulating VES-H_8_R_8_ and pDox to maximize synergism. (*** <0.001) The increase in ROS upon VDNPs treatment is consistent with the oxidative stress typically observed with both Dox and VES-H_8_R_8_ treatment^14,51^. It is clear that mitochondrial membrane depolarization and induction of ROS is involved in the synergistic cytotoxicity of VDNPs.

### Doubly loaded NPs induce mainly apoptosis

**Figure 5:**
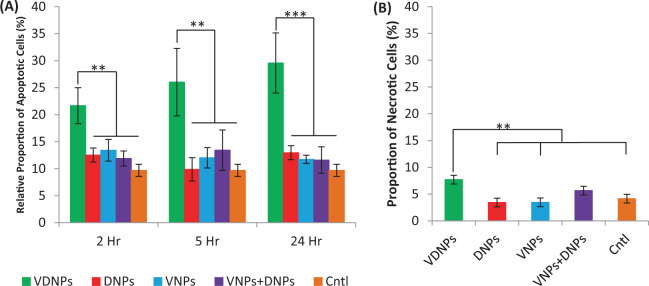
Vitamin E succinate modified octahistidine-octaarginine and palmityl-doxoribicin NPs (VDNPs) induce apoptosis in a time-dependent manner and necrosis after a 24 h treatment. (**A**) Only VDNPs induced apoptosis in a time-dependent manner whereas singly-loaded NPs, palmityl-doxoribicin NPs (DNPs) and vitamin E succinate modified octahistidine-octaarginine NPs (VNPs), or their mixture (VNPs + DNPs) did not induce apoptosis at 2, 5, and 24 h of incubation. (**B**) VDNPs increased the proportion of cells that were necrotic after a 24 h incubation, relative to singly-loaded NPs, DNPs and VNPs, or their mixture (VNPs + DNPs). VDNPs were incubated at the IC_50_, where the other NP formulations were dose-matched to the respective drug concentration for either palmityl-doxorubicin (pDox) or vitamin E succinate modified octahistidine-octaarginine (VES-H_8_R_8_). Data are presented as a mean (n = 3) ± SD, and statistical analysis was performed using one-way ANOVA and Tukey’s multiple comparison test (** <0.01, *** <0.001).

Mitochondrial membrane depolarization and induction of ROS typically result in apoptosis^52^. Furthermore, VES-H_8_R_8_ was previously shown to induce apoptosis in the MDR EMT6/AR-1 cells^14^. To avoid fluorescent bleed over from Dox, apoptotic and necrotic cells were stained separately with Annexin V-Cy5 and 7-AAD. VDNPs incubated at its IC_50_ significantly increased the proportion of apoptotic cells at 2, 5, and 24 h of incubation relative to singly-loaded NPs, DNPs and VNPs, and their mixture (** <0.01) (Fig. 5A). Specifically, VDNPs increased apoptotic levels to 21.7 ± 3.3%, 26.0 ± 6.3%, and 29.6 ± 5.6% upon incubation at 2, 5, and 24 h, respectively. Importantly, co-treating cells simultaneously with DNPs and VNPs did not significantly induce apoptosis relative to no treatment control group and both singly loaded NPs, further supporting the required co-encapsulation to trigger apoptosis. Thus, for annexin V-Cy5 binding, VDNP treatment likely resulted in EMT6/AR-1 cells exposing phosphatidylserine on the cell surface membrane. The induction of apoptosis by VDNPs agrees with the induced apoptosis observed with the treatment of EMT6/AR-1 cells with VES-H_8_R_8_, as previously shown^14^. As necrotic cells with compromised membranes could also stain for annexin-V, as a false positive for apoptosis, we evaluated the induction of necrosis upon VDNPs treatment. VDNPs significantly increased the proportion of necrotic cells relative to no treatment control (** <0.01) (Fig. 5B). However, the increase in necrotic cell number was not as large as the increase in the proportion of apoptotic cells, suggesting that VDNPs treatment causes minimal membrane damage, and therefore mainly induces apoptosis. This is consistent with the initial induction of apoptosis by doxorubicin on Jurkat cells after a 24 h treatment, followed by the induction of necrosis after a 60 h treatment^53^.

## Conclusion

We demonstrated, for the first time, the synergistic effect of VES-H_8_R_8_ and pDox co-encapsulation against MDR EMT6/AR-1 cells. We showed that co-encapsulation is superior to treatment with VNPs and DNPs together. Our investigation suggests that the synergistic mechanism involves increased cell uptake, enhanced Dox retention, Pgp inhibition, mitochondrial membrane depolarization, induction of ROS, and mainly apoptosis. This study highlights the significance of co-encapsulating a novel mitochondria depolarizer and Pgp efflux inhibitor, VES-H_8_R_8_, with a pH-sensitive prodrug of Dox in NPs to synergistically kill MDR breast cancer cells *in vitro*.

## Supplementary information

Supplementary Information.
