# Supplementary material for: Nanoparticle delivery of a pH-sensitive prodrug of doxorubicin and a mitochondrial targeting VES-H8R8 synergistically kill multi-drug resistant breast cancer cells
Source: Sci Rep. 2020 May 26;10:8726. doi: 10.1038/s41598-020-65450-x (PMC7251113; doi:10.1038/s41598-020-65450-x)
Supplement: Supplementary file 1 — Supplementary Information. [file 41598_2020_65450_MOESM1_ESM.pdf]

Nanoparticle delivery of a pH-sensitive prodrug of doxorubicin and a mitochondrial targeting VES-H_8R_8 synergistically kill multi-drug resistant breast cancer cells.

Petro Czupiel[a,b,c,], Vianney Delplace[a,b,c], Molly Shoichet[a,b,c]

[a]Department of Chemical Engineering and Applied Chemistry, University of Toronto, 200 College Street, Toronto, ON, M5S 3E5, Canada
[b]Institute of Biomaterials and Biomedical Engineering, University of Toronto, 164 College Street, Toronto, ON, M5S 3G9, Canada
[c]Donnelly Centre, University of Toronto, 160 College Street, Toronto, ON, M5S 3E1, Canada

*Corresponding author. Email: molly.shoichet@utoronto.ca

<u>**Supplementary Information**</u>

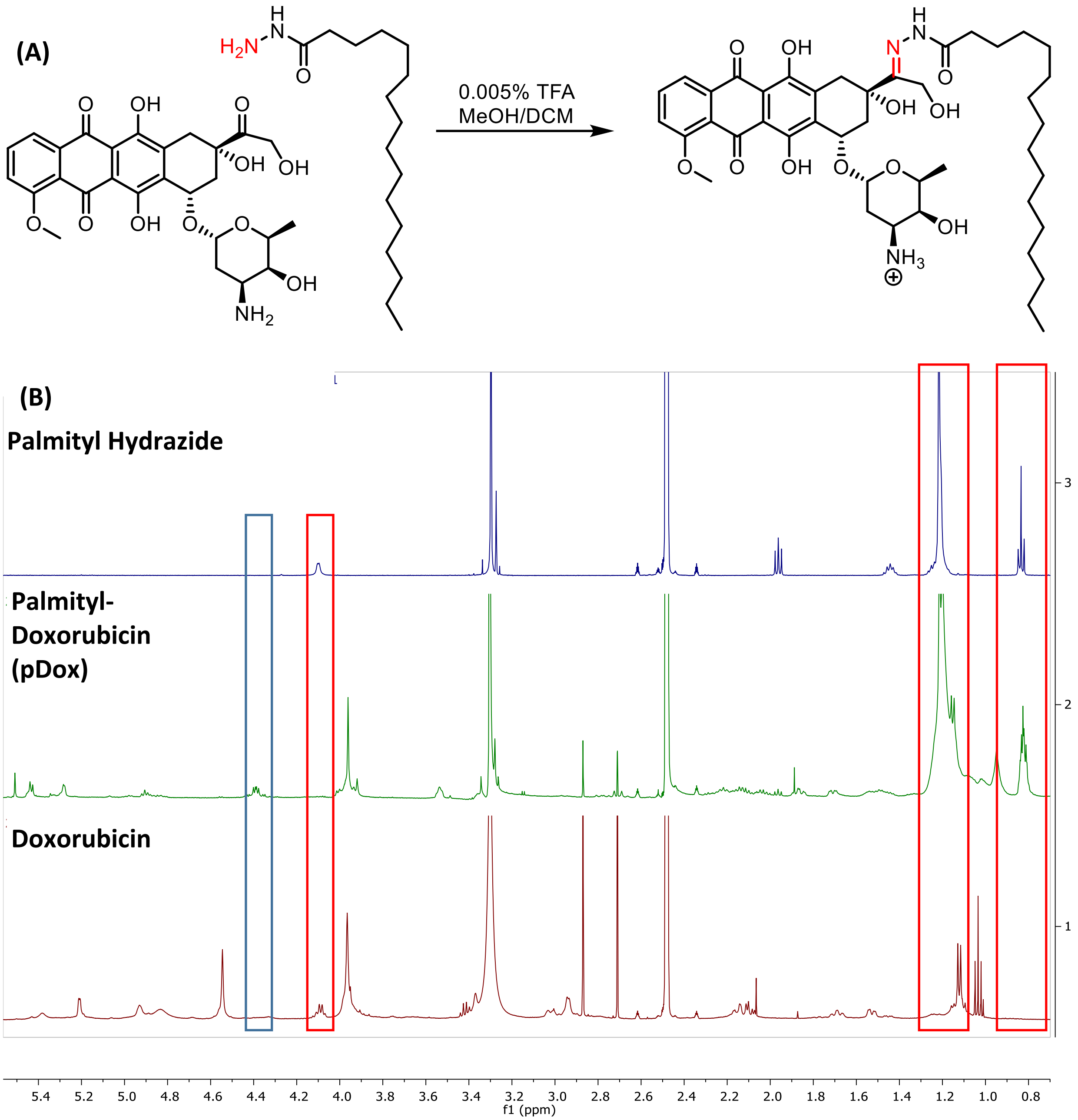

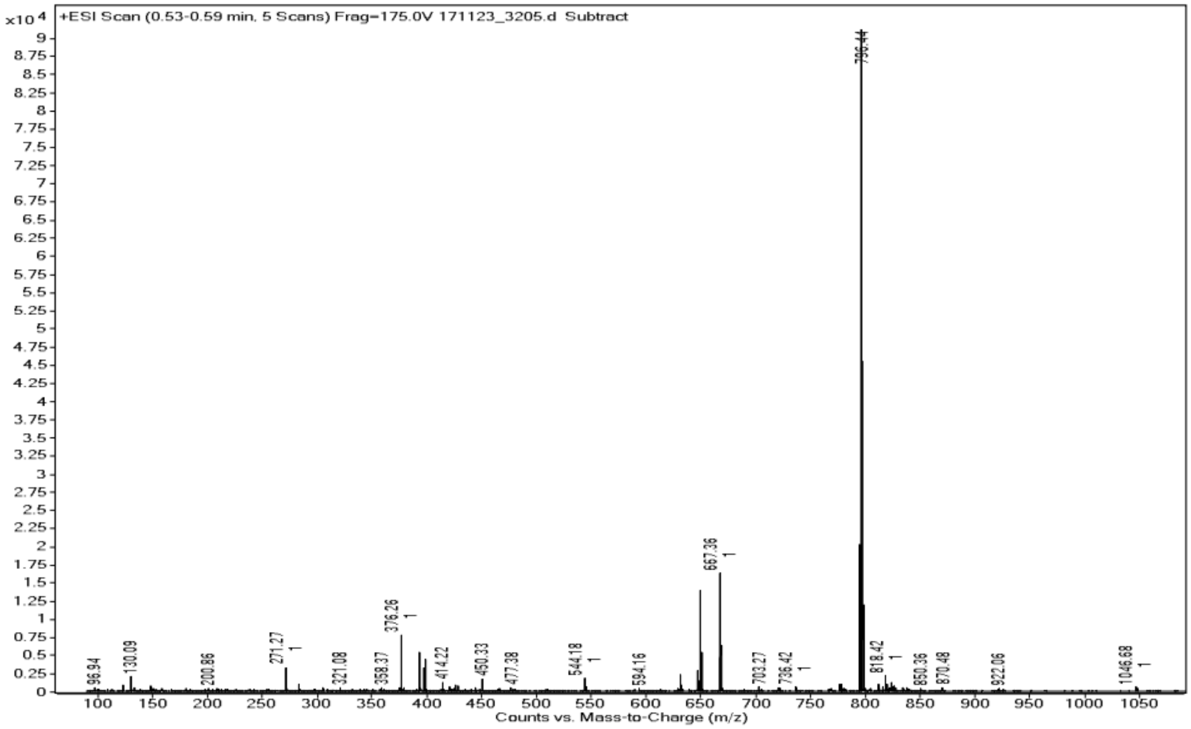

Figure S1 Synthesis and characterization of pH-responsive palmityl-doxorubicin (pDox). (A) Schematic representing the sy nthesis of pDox using trifluoroacetic acid (TFA) as a catalyst in a dichloromethane/methanol solution. (B) Nuclear magnetic resonance (NMR) spectra of palmityl hydrazide, palmityl-doxorubicin (pDox), and doxorubicin (top to bottom). The red boxes represent the H^1 peaks attributed from palmitic hydrazide, while the blue box represents validification of the hydrazone bond formation in pDox. (C) Mass spectrometry results of purified (pDox) confirming successful synthesis (expected: 796.63 g/mol; obtained: 796.44 g/mol).

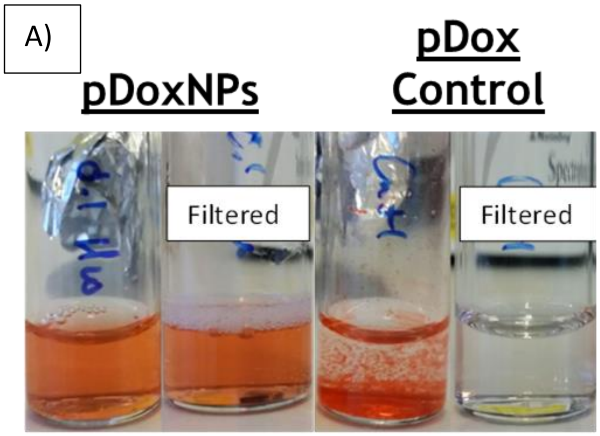

B)

Name	[pDox] (μM)	[VES-H_8R_8] (μM)
DNPs	581.4 ±69.4	0.0
VNPs	0.0	185.4 ± 11.1
VDNPs	386.5 ± 46.1	141.6 ± 8.4

Figure S2 A) Palmityl-doxorubicin (pDox) mixed with poly(D,L-lactide-co-2-methyl-2-carboxytrimethylenecarbonate)$_{12K}$-grafted-poly(ethylene glycol)$_{10K}$-azide [P(LA-*co*-TMCC)-*g*-PEG-N_3] spontaneously forms nanoparticles (NPs) that can be filtered for in vitrp/in vivo investigation. pDox alone immediately precipitates and gets filtered off completely. B) Concentration of each drug in the encapsulated nanoparticles.

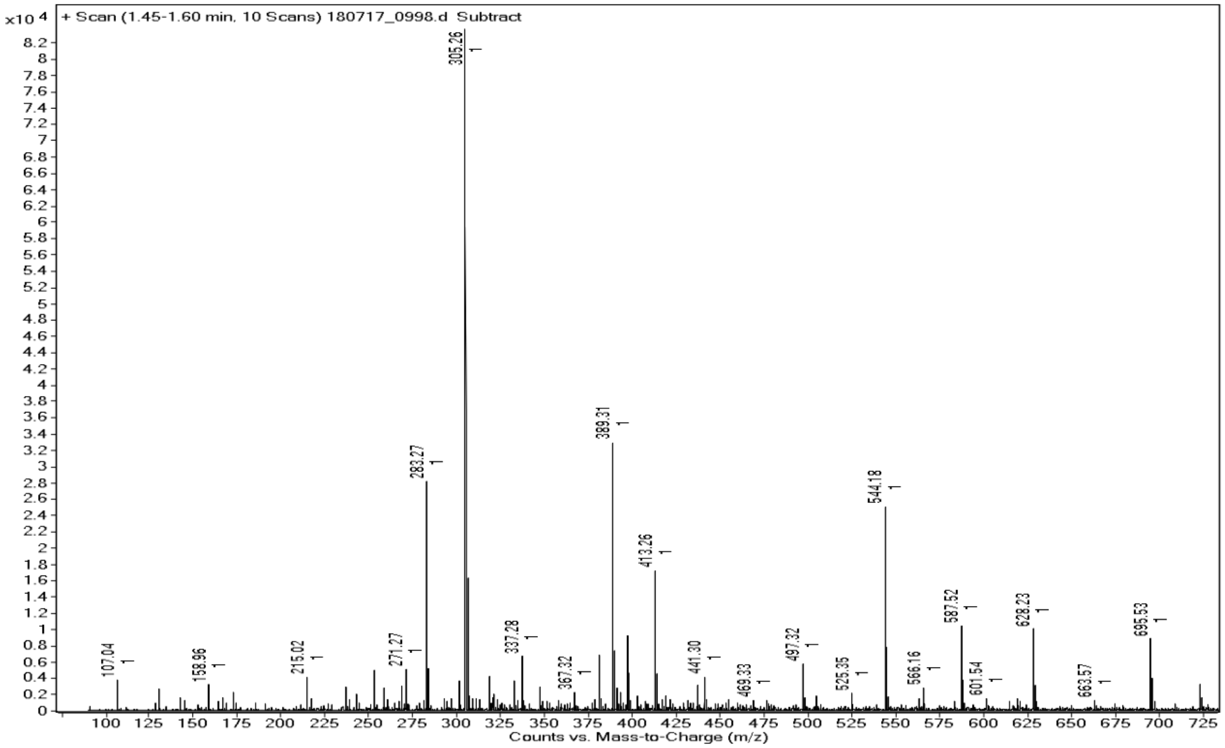

Figure S3 Characterization of the pH-responsive release of doxorubicin from palmityl-doxorubicin (pDox) by mass spectrometry (expected: 544.17 g/mol; obtained: 544.18 g/mol) when pDox was incubated in 1:1 DMSO:PBS (pH 5.0) at 37 °C for 24 h. Free palmitic hydrazide is observed at 271.27 g/moL (expected: 270.46 g/mol) while molecular ion adducts are observed at 305.26 g/mol (M+CH3OH+H).

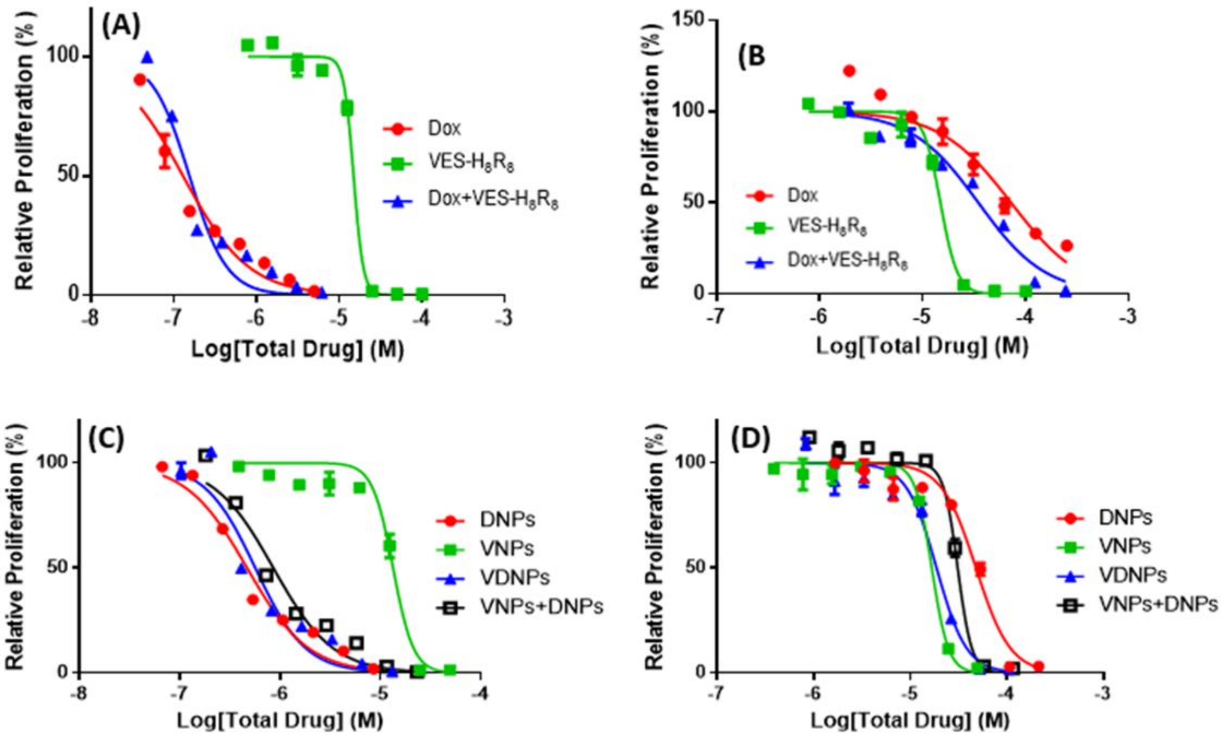

Figure S4. Dose response curves of free drugs in EMT6/P (A), and EMT6/AR-1 (B), and those of doubly-loaded nanoparticles (VDNPs) in EMT6/P (C) and EMT6/AR-1 (D). Dose response curves were obtained after a 24 h incubation, followed by 48 h in fresh medium to allow cells to grow. Singly-loaded NPs, palmityl-doxorubicin NPs (DNPs) and vitamin E succinate modified octahistidine-octaarginine NPS (VNPs), doubly-loaded NPs, vitamin E succinate modified octahistidine-octaarginine-pDox NPs (VDNPS), and vitamin E succinate modified octahistidine-octaarginine (**VES-H_8R_8**) were tested for anti-cancer activity. Presto blue analysis was used to evaluate the relative cell proliferation.

Table S1. IC_{50} and combination indices of free drugs and drug-loaded nanoparticles (NPs), in the parental breast cancer cell line, EMT6/P.

**Name**	**Dox:VES-H_8R_8 Molar Ratio**[1]	**IC_{50} (µM, Total drug)**[2]	**Combination Index**[3]	**Dox Dose Reduction Index**[3]	**VES-H_8R_8 Dose Reduction Index**[3]
Dox		0.16 ± 0.02			
VES-H_8R_8		11.12 ± 2.56			
Dox+ VES-H_8R_8	1 : 0.37	0.30 ± 0.03	1.51 ± 0.08	0.65 ± 0.03	224.59 ± 63.56
DNPs	1 : 0	0.68 ± 0.12			
VNPs	0 : 1	8.34 ± 1.45			
DNPs + VNPs	1 : 0.37	0.99 ± 0.10	0.99 ± 0.10	0.95 ±0.24	
VDNPs	1 : 0.37	0.97 ± 0.14	0.96 ± 0.14	0.97 ± 0.15	

[1][Dox] calculated using fluorescence and [VES-H_8R_8] calculated using amino acid analysis.
[2]Obtained from Presto Blue analysis of drug treated cells.
[3]Calculated using CompuSyn.

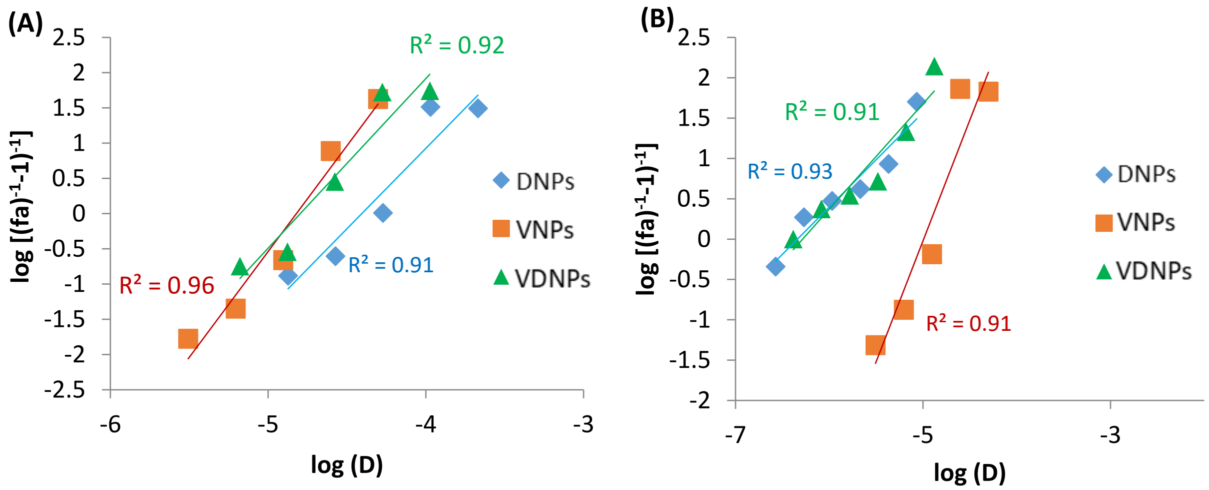

Figure S5. Linearized dose response curves of palmityl-doxorubicin-NPs (DNPs), vitamin E succinate modified octahistidine-octaarginine-NPs (VNPs), and co-encapsulated vitamin E succinate modified octahistidine-octaarginine and palmityl-doxorubicin (VDNPs), in EMT6/-AR-1 cells (A) and EMT6/P cells (B). fa represents the fraction of dead cells and D is the total drug concentration in each formulation. Non-parallel lines suggest non-exclusivity in the mechanism of action of VNPs and DNPs. R^2 values greater than 0.9 indicate statistical validity of the analysis and conforms to mass action law.

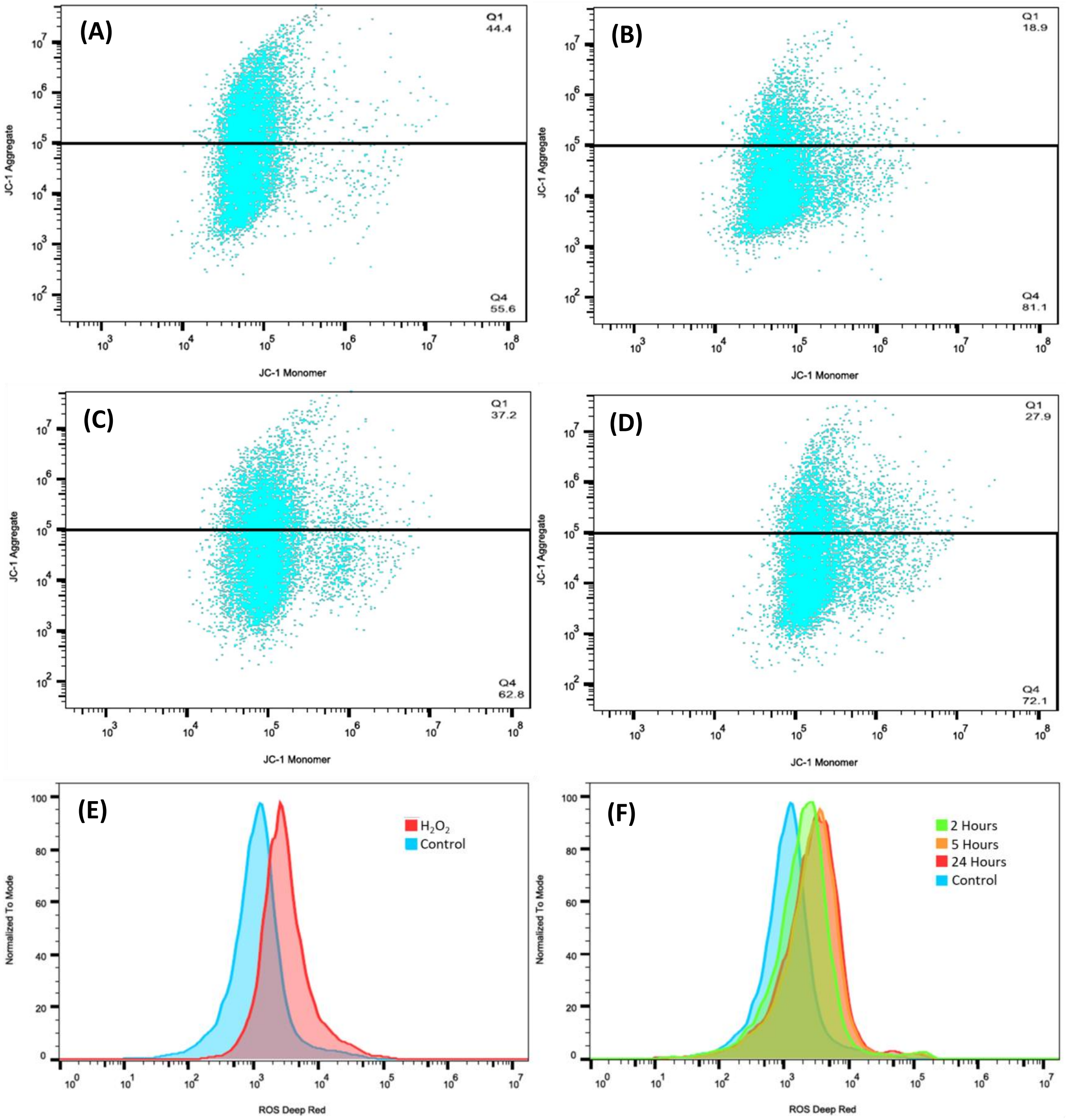

(A)
Q1
44.4
Q4
55.6
JC-1 Aggregate
JC-1 Monomer
(B)
Q1
18.9
Q4
81.1
JC-1 Aggregate
JC-1 Monomer
(C)
Q1
37.2
Q4
62.8
JC-1 Aggregate
JC-1 Monomer
(D)
Q1
27.9
Q4
72.1
JC-1 Aggregate
JC-1 Monomer
(E)
H_2O_2
Control
Normalized To Mode
ROS Deep Red
(F)
2 Hours
5 Hours
24 Hours
Control
Normalized To Mode
ROS Deep Red

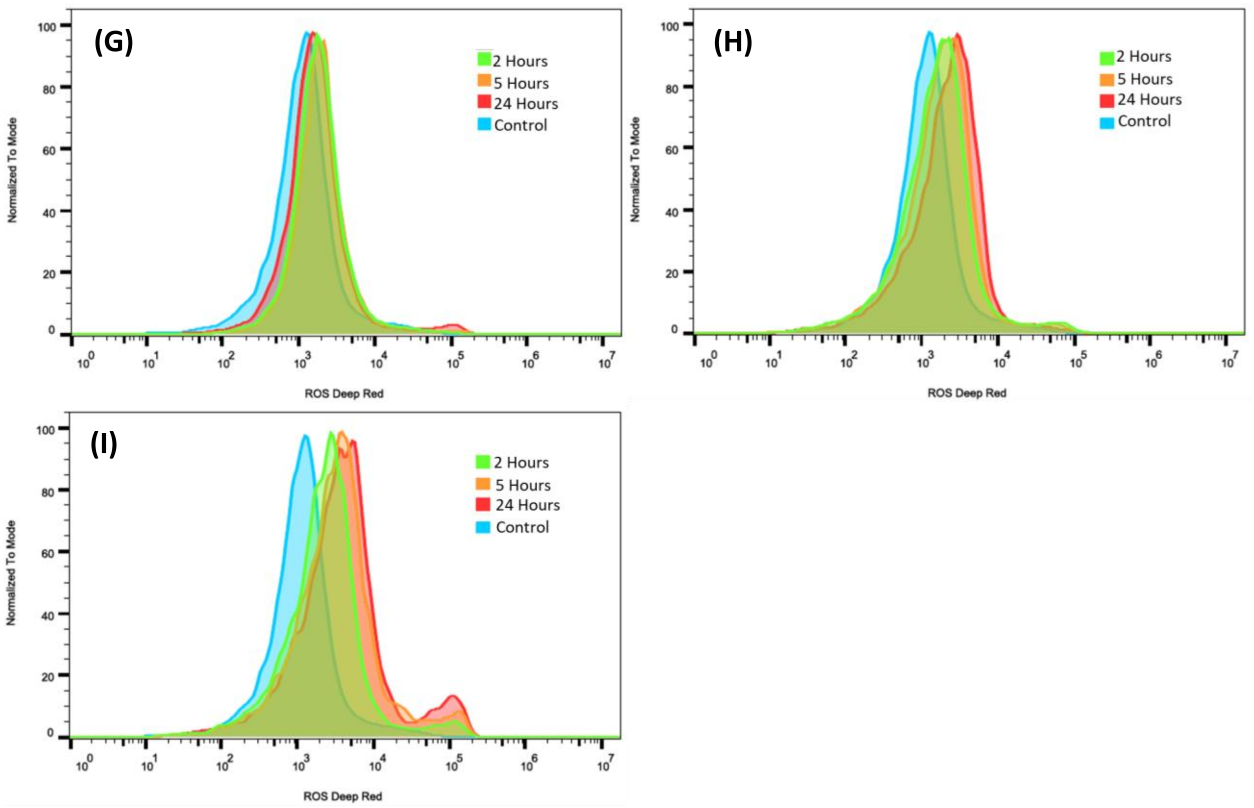

Figure S6. Representative flow cytometry images for the data presented in Figure 5. Representative flow cytometry images are shown for the JC-1 measurement after a treatment of (A) no treatment control, (B) negative control CCCP, (C) VNPs at 4 µM and (D) VNPs at 14 µM. The x-axis represents the JC-1 monomer in the FL1 channel (ex = 488, em = 533/30 nm) and the y-axis represents the JC-1 aggregate in the FL2 channel (ex. = 488 nm, em. = (585/40 nm). CCCP = Carbonyl cyanide 3-chlorophenylhydrazone, mitochondrial membrane potential disruptor Representative flow cytometry images of the (E) induced ROS as measured by the ROS deep red probe in the H_2O_2 positive control; and cells treated with (F) DNPs, (G) VNPs, (H) DNPs+VNPs, and (I) VDNPs.
